# MicroRNA-277 Modulates the Neurodegeneration Caused by Fragile X Premutation rCGG Repeats

**DOI:** 10.1371/journal.pgen.1002681

**Published:** 2012-05-03

**Authors:** Huiping Tan, Mickael Poidevin, He Li, Dahua Chen, Peng Jin

**Affiliations:** 1 Department of Human Genetics, Emory University School of Medicine, Atlanta, Georgia, United States of America; 2 Division of Histology and Embryology, Tongji Medical College, Huazhong University of Science and Technology, Wuhan, China; 3 State Key Laboratory of Reproductive Biology, Institute of Zoology, Chinese Academy of Sciences, Beijing, China; University of Minnesota, United States of America

## Abstract

Fragile X-associated tremor/ataxia syndrome (FXTAS), a late-onset neurodegenerative disorder, has been recognized in older male fragile X premutation carriers and is uncoupled from fragile X syndrome. Using a *Drosophila* model of FXTAS, we previously showed that transcribed premutation repeats alone are sufficient to cause neurodegeneration. MiRNAs are sequence-specific regulators of post-transcriptional gene expression. To determine the role of miRNAs in rCGG repeat-mediated neurodegeneration, we profiled miRNA expression and identified selective miRNAs, including miR-277, that are altered specifically in *Drosophila* brains expressing rCGG repeats. We tested their genetic interactions with rCGG repeats and found that miR-277 can modulate rCGG repeat-mediated neurodegeneration. Furthermore, we identified Drep-2 and Vimar as functional targets of miR-277 that could modulate rCGG repeat-mediated neurodegeneration. Finally, we found that hnRNP A2/B1, an rCGG repeat-binding protein, can directly regulate the expression of miR-277. These results suggest that sequestration of specific rCGG repeat-binding proteins could lead to aberrant expression of selective miRNAs, which may modulate the pathogenesis of FXTAS by post-transcriptionally regulating the expression of specific mRNAs involved in FXTAS.

## Author Summary

Fragile X–associated tremor/ataxia syndrome (FXTAS) is an adult-onset neurodegenerative disorder, usually affecting males over 50 years of age. FXTAS patients are the carriers of fragile X premutation alleles. Using a FXTAS *Drosophila* model, we previously demonstrated that fragile X premutation rCGG repeats alone could cause neurodegeneration. Pur α and hnRNP A2/B1 were identified as specific premutation rCGG repeat-binding proteins (RBPs) that could bind and modulate fragile X permutation rCGG-mediated neuronal degeneration. MiRNAs are sequence-specific regulators of post-transcriptional gene expression. Here we show that fragile X premutation rCGG repeats could lead to aberrant expression of selective miRNAs, which may modulate the pathogenesis of FXTAS by post-transcriptionally regulating the expression of specific mRNAs involved in FXTAS.

## Introduction

Fragile X syndrome (FXS), the most common form of inherited mental retardation, is caused by expansion of the rCGG trinucleotide repeat in the 5′ untranslated region (5′ UTR) of the fragile X mental retardation 1 (*FMR1*) gene, which leads to silencing of its transcript and the loss of the encoded fragile X mental retardation protein (FMRP) [1]–[6]. Most affected individuals have more than 200 rCGG repeats, referred to as full mutation alleles [7]. Fragile X syndrome carriers have FMR1 alleles, called premutations, with an intermediate number of rCGG repeats between patients (>200 repeats) and normal individuals (<60 repeats) [8]. Recently, the discovery was made that male and, to a lesser degree, female premutation carriers are at greater risk of developing an age-dependent progressive intention tremor and ataxia syndrome, which is uncoupled from fragile X syndrome and known as fragile X-associated tremor/ataxia syndrome (FXTAS) [9], [10]. This is combined with cognitive decline associated with the accumulation of ubiquitin-positive intranuclear inclusions broadly distributed throughout the brain in neurons, astrocytes, and in the spinal column [11], [12].

At the molecular level, the premutation is different from either the normal or full mutation alleles. Based on the observation of significantly elevated levels of rCGG-containing FMR1 mRNA, along with either no detectable change in FMRP or slightly reduced FMRP levels in premutation carriers, an RNA-mediated gain-of-function toxicity model has been proposed for FXTAS [13]–[17]. Several lines of evidence in mouse and *Drosophila* models further support the notion that transcription of the CGG repeats leads to this RNA-mediated neurodegenerative disease [11], [15], [17]–[19]. The hypothesis is that specific RNA-binding proteins may be sequestered by overproduced rCGG repeats in FXTAS and become functionally limited, thereby contributing to the pathogenesis of this disorder [15], [17], [19], [20]. There are three RNA-binding proteins found to modulate rCGG-mediated neuronal toxicity: Pur α, hnRNP A2/B1, and CUGBP1, which bind rCGG repeats either directly (Pur α and hnRNP A2/B1) or indirectly (CUGBP1, through the interaction with hnRNP A2/B1) [21], [22].

MicroRNAs (miRNAs) are small, noncoding RNAs that regulate gene expression at the post-transcriptional level by targeting mRNAs, leading to translational inhibition, cleavage of the target mRNAs or mRNA decapping/deadenylation [23], [24]. Mounting evidence suggests that miRNAs play essential functions in multiple biological pathways and diseases, from developmental timing, fate determination, apoptosis, and metabolism to immune response and tumorigenesis [25]–[31]. Recent studies have shown that miRNAs are highly expressed in the central nervous system (CNS), and some miRNAs have been implicated in neurogenesis and brain development [32]–[34].

Interest in the functions of miRNAs in the CNS has recently expanded to encompass their roles in neurodegeneration. Investigators have begun to reveal the influence of miRNAs on both neuronal survival and the accumulation of toxic proteins that are associated with neurodegeneration, and are uncovering clues as to how these toxic proteins can influence miRNA expression [35]. For example, miR-133b is found to regulate the maturation and function of midbrain dopaminergic neurons (DNs) within a negative feedback circuit that includes the homeodomain transcription factor Pitx3 in Parkinson's disease [36]. In addition, reduced miR-29a/b-1-mediated suppression of BACE1 protein expression contributes to Aβ accumulation and Alzheimer's disease pathology [37]. Moreover, the miRNA *bantam* is found to be a potent modulator of poly-Q- and tau-associated degeneration in *Drosophila* [38]. Other specific miRNAs have also been linked to other neurodegenerative disorders, such as spinocerebellar ataxia type 1 (SCA1) and Huntington's disease (HD) [39], [40]. Therefore, miRNA-mediated gene regulation could be a novel mechanism, adding a new dimension to the pathogenesis of neurodegenerative disorders.

Here we show that fragile X premutation rCGG repeats can alter the expression of specific miRNAs, including miR-277, in a FXTAS *Drosophila* model. We demonstrate that miR-277 modulates rCGG-mediated neurodegeneration. Furthermore, we identified Drep-2, which is associated with the chromatin condensation and DNA fragmentation events of apoptosis, and Vimar, a modulator of mitochondrial function, as two of the mRNA targets regulated by miR-277. Functionally, Drep-2 and Vimar could modulate the rCGG-mediated neurodegeneration, as well. Finally, we show that hnRNP A2/B1, an rCGG repeat-binding protein, can directly regulate the expression of miR-277. These data suggest that hnRNP A2/B1 could be involved in the transcriptional regulation of selective miRNAs, and fragile X premutation rCGG repeats could alter the expression of specific miRNAs, potentially contributing to the molecular pathogenesis of FXTAS.

## Results

### Fragile X premutation rCGG repeats alter the expression of selective miRNAs

**Figure 1:**
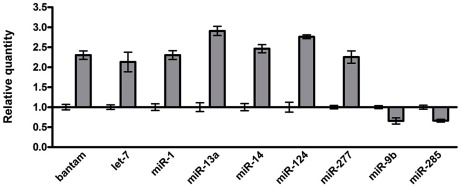
Identification of the miRNAs with altered expression in the brains of an FXTAS *Drosophila* model. Relative quantity of miRNA with ≥two-fold change in expression shown for rCGG_60_ flies, calibrated to control (elav-GAL4) flies. Control relative quantity = 1.

Given the important roles of miRNAs in neural development and human neurological disorders, we investigated the role of miRNAs in rCGG-mediated neurodegeneration. To determine whether fragile X premutation rCGG repeats could influence the expression of miRNAs, we profiled the expression of 72 known miRNAs using rCGG repeat transgenic flies that we generated previously [15]. In rCGG repeat transgenic flies, the severity of their phenotype depends on both dosage and length of the rCGG repeat. Moderate expression of (CGG)_90_ repeats exclusively in the eyes have an effect on morphology and histology; however, expression of (CGG)_90_ repeats in the neurons leads to lethality at the embryonic stage, preventing analysis at the adult stage [15]. Therefore, we used a shorter repeat length, r(CGG)_60_, which allowed us to examine the gene expression in adults. To analyze the effect of rCGG repeats in adult brains, we used RNAs isolated from the age- and sex-matched brains of control flies (*elav-GAL4*) and flies expressing rCGG_60_ repeats in neurons (*elav-GAL4;UAS-CGG_60_-EGFP*) for miRNA profiling experiments (Figure 1). We identified a subset of miRNAs that consistently displayed altered expression in rCGG repeat flies versus the control group. Seven miRNAs with a ≥two-fold increase and two miRNAs with expression decreased by ≥1.5-fold have been found in rCGG repeat flies. These results suggest that fragile X premutation rCGG repeats could lead to the dysregulation of a subset of specific miRNAs.

### Overexpression of miR-277 enhances rCGG-mediated neurodegeneration

**Figure 2:**
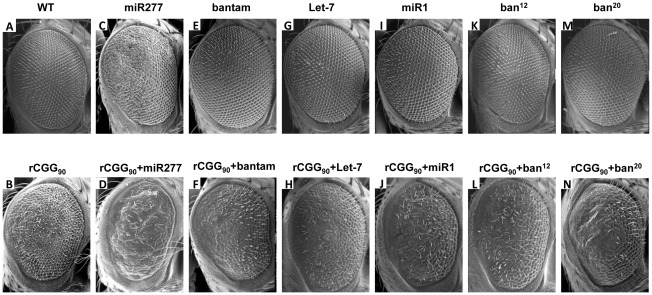
Overexpression of miR-277 enhances rCGG-mediated neurodegeneration. Shown are scanning electron microscope (SEM) eye images from seven-day-old flies. WT fly eyes show the normal organization of ommatidia (A). Expression of rCGG90 causes disorganized, fused ommatidia (B). Flies overexpressing miR-277 alone present with a mild rough eye phenotype (C). The rCGG90 eye phenotype is enhanced by miR-277 overexpression with aggravated disorganized, fused ommatidia (D). Alteration of bantam, let-7, or miR-1 does not modify the rCGG-induced eye phenotype (F, H, J, L, N). Alteration of bantam, let-7 or miR-1 alone does not cause an abnormal eye phenotype (E, G, I, K, M). Genotypes are B*-gmr-GAL4,UAS-CGG_90_-EGFP/+*; C*-gmr-GAL4/UAS-miR-277*; D*-gmr-GAL4, UAS-CGG_90_-EGFP/UAS-miR-277*; E-*gmr-GAL4/+;UAS-bantam/+*; F-*gmr-GAL4,UAS-CGG_90_-EGFP/+;UAS-bantam/+*; G-*gmr-GAL4/+;UAS-Let-7/+*; H-*gmr-GAL4,UAS-CGG_90_-EGFP/+;UAS-Let-7/+*; I-*gmr-GAL4/UAS-GFP;UAS-miR-1/+*; J-*gmr-GAL4,UAS-CGG_90_-EGFP/UAS-GFP;UAS-miR-1/+*; K-*gmr-GAL4/+;ban^12^/+*; L-*gmr-GAL4,UAS-CGG_90_-EGFP/+;ban^12^/+*; M-*gmr-GAL4/+;ban^20^/+*; N-*gmr-GAL4,UAS-CGG_90_-EGFP/+;ban^20^/+*.

To assess the potential involvement of the miRNAs that showed altered expression in FXTAS fly brain, we examined the genetic interaction between specific miRNAs and rCGG-mediated neuronal toxicity based on the fragile X premutation rCGG repeat-mediated neurodegenerative eye phenotype we observed previously [15]. We generated *UAS* fly lines that could overexpress *Drosophila* miR-277, bantam, let-7, or miR-1, as well as bantam mutant lines (*ban^12^* and *ban^20^*) that we generated previously [41]. We then crossed these transgenic lines with *gmr-GAL4, UAS-(CGG)_90_-EGFP* transgenic flies that exhibit photoreceptor neurodegeneration to determine the role of specific miRNAs in rCGG-mediated neurodegeneration. As shown in Figure 2, flies co-expressing miR-277 and rCGG_90_ consistently showed an aggravated eye phenotype, with enhanced disorganized, fused ommatidia compared with flies expressing rCGG_90_ alone (Figure 2B and 2D). Flies overexpressing miR-277 alone displayed a very mild rough eye phenotype (Figure 2C). Alterations of the levels of bantam, let-7, or miR-1 by either a gain of function or loss of function had no effect on rCGG-mediated neurodegeneration (Figure 2E–2N). These data together suggest that miR-277 could be involved in rCGG-mediated neurodegeneration. The role of miR-277 in rCGG-mediated neurodegeneration seems specific, since the other miRNAs we found with altered expression in the presence of fragile X premutation rCGG repeats, including bantam, let-7, and miR-1, had no effect on the rCGG_90_ eye phenotype. The rest of our work focused on the role of miR-277 and its potential mechanisms in modulating rCGG-mediated neurodegeneration.

### Blocking the activity of miR-277 suppresses rCGG-mediated neurodegeneration

**Figure 3:**
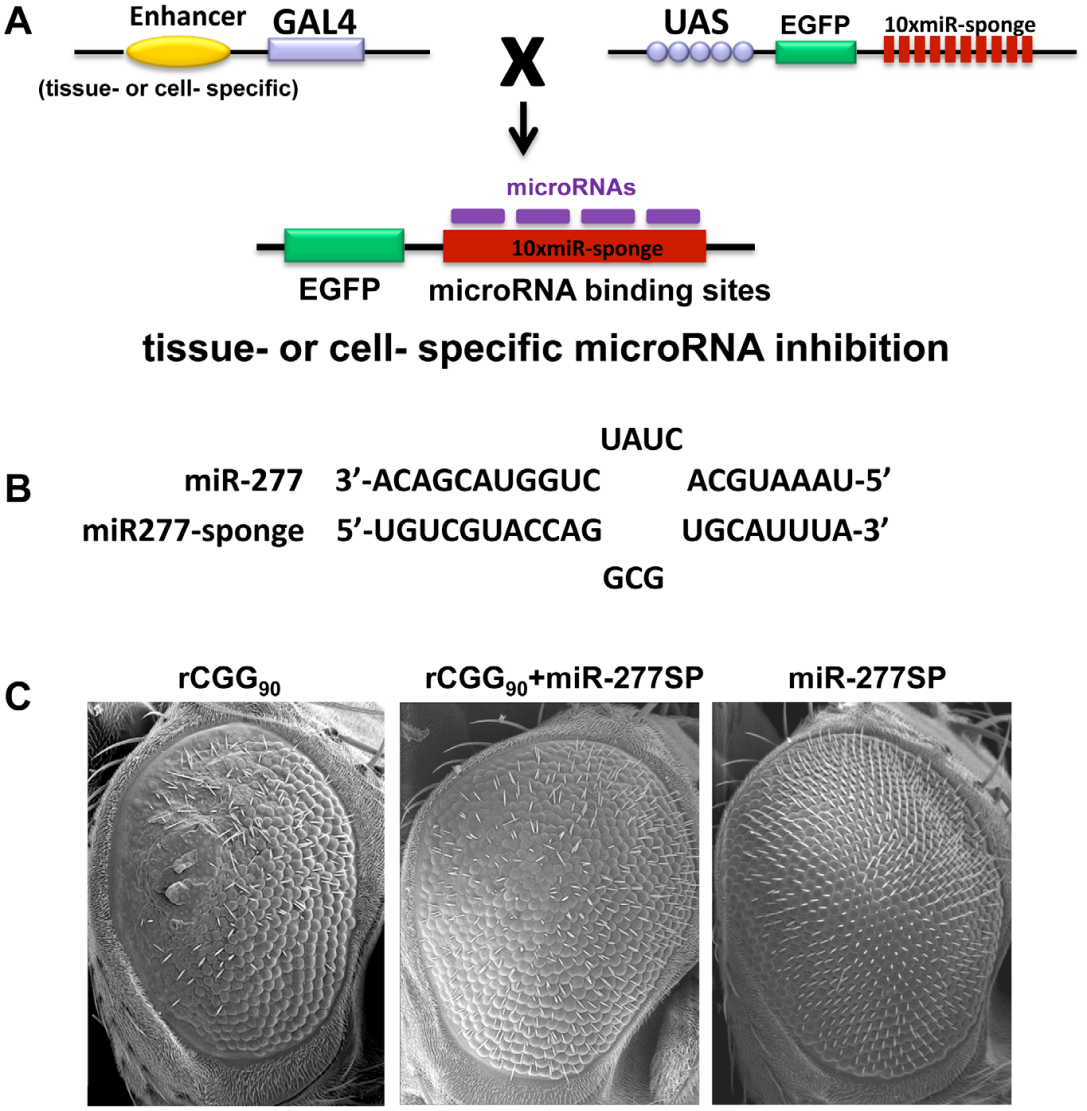
Blocking the activity of miR-277 suppresses rCGG-mediated neurodegeneration. A. Shown is the diagram depicting the strategy for generation of transgenic microRNA sponge lines. The sponge construct contains 10 repetitive sequences complementary to a microRNA downstream of EGFP in a UAS-containing P-element vector. When these UAS lines are crossed to GAL4 driver lines, the F1 progeny generate tissue- or cell-specific expression of microRNA sponge to block the activity of specific miRNA in *Drosophila*. B. The miR-sponge contains bulged sites that are mismatched opposite microRNA at positions 9–12. The bulge is designed to protect Ago2-mediated endonucleolytic cleavage. C. Scanning electron microscope (SEM) eye images from seven-day-old flies. Block of miR-277 activity by miR-277SP could rescue the rCGG-mediated neurodegenerative eye phenotype. Knockdown of miR-277 alone does not cause an abnormal eye phenotype. Genotypes are (left)*-gmr-GAL4,UAS-CGG_90_-EGFP/+*; (middle)*-gmr-GAL4,UAS-CGG_90_-EGFP/UAS-miR-277SP*; (right)-*gmr-GAL4/UAS-miR-277SP*.

Our miRNA profiling and genetic interaction studies indicated that an increase in miR-277 expression in rCGG repeat flies could alter the expression of specific cellular mRNAs by miR-277, resulting in the enhanced rCGG-induced eye phenotype. To further explore the potential regulatory effect of miR-277 on rCGG-mediated neurodegeneration, we generated a transgenic miR-277 sponge (miR-277SP) line, which could block the activity of miR-277, to test for any blocking effect on the rCGG-induced neurodegenerative eye phenotype. We generated the miRNA sponge transgenic construct as described previously [42], [43]. In brief, we placed 10 repetitive sequences complementary to miR-277 with mismatches at positions 9–12 into the 3′ UTR of EGFP in a pUASP expression vector (Figure 3A). We crossed miR-277SP transgenic flies with the flies expressing 90 CGG repeats and found that the expression of miR-277 sponge could consistently suppress rCGG-mediated neuronal toxicity (Figure 3B). MiR-277 sponge alone or scramble control sponge had no effect on eye morphology (Figure 3B and Data not shown). This result suggests that blocking the activity of miR-277 could mitigate the neurodegeneration caused by fragile X premutation rCGG repeats.

### Identification of miR-277 mRNA targets that modulate rCGG-mediated neurodegeneration

**Figure 4:**
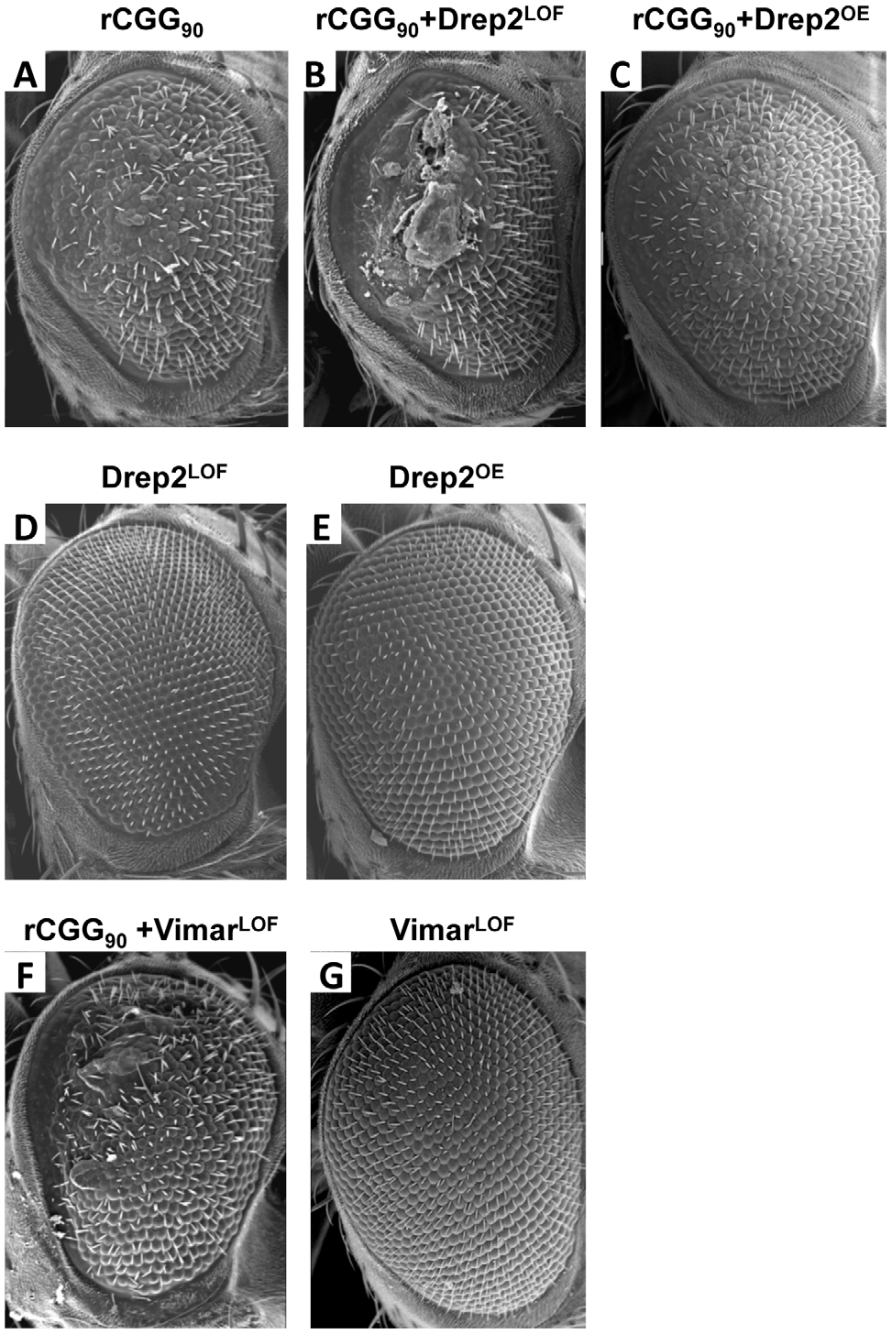
Modification of rCGG-mediated neurodegenerative eye phenotype by Drep-2 and Vimar. (A–C) SEM eye images from control flies expressing rCGG_90_, rCGG_90_ and a heterozygous mutation in Drep-2 (Drep-2^LOF^), rCGG_90_ and overexpression of Drep-2 (Drep-2^OE^), and rCGG_90_ and a heterozygous mutation in Vimar (Vimar^LOF^). rCGG_90_ flies manifest ommatidial disorganization and fusion (A). This phenotype is enhanced in flies carrying a heterozygous loss-of-function mutation in Drep-2 (B), while the eye phenotype could be suppressed by overexpression of Drep-2 (C). Mutation of Vimar enhances the rCGG_90_-mediated eye phenotype (F). All the mutants alone do not cause any abnormal eye phenotype (D, E, G). Genotypes are A-*gmr-GAL4,UAS-CGG_90_-EGFP/+*; B -*gmr-GAL4, UAS-CGG_90_-EGFP/Drep-2^KG02396^*; C-*gmr-GAL4, UAS-CGG_90_-EGFP/Drep-2^d00223^*; D-*gmr-GAL4/Drep-2^KG02396^*; E-*gmr-GAL4/ Drep-2^d00223^*; F -*gmr-GAL4, UAS-CGG_90_-EGFP/ Vimar^K16722^*; G-*gmr-GAL4/ Vimar^K16722^*.

To seek the mechanisms by which miR-277 modulates rCGG-mediated neurodegeneration, we searched the RNA network and referenced TargetScanFly 5.1 to identify potential miR-277 targets [44], [45]. We selected the top candidates for miR-277 target mRNAs with the mutant alleles available for further analyses (Table 1). We then carried out a genetic screen on the rCGG_90_ neurodegenerative eye phenotype to identify potential miR-277 targets that could modulate rCGG-mediated neuronal toxicity. We crossed *gmr-GAL4, UAS-(CGG)_90_-EGFP* transgenic flies with fly mutants in genes coding for the top candidates for miR-277 target genes (Table 1). The progenies were then tested for potential suppression or enhancement of the disorganized eye phenotype versus flies expressing rCGG_90_ alone. Through this screen, we identified two modifiers of rCGG-mediated neurodegeneration, Drep-2 and Vimar. As shown in Figure 4, partial loss of Drep-2 could enhance the rCGG_90_ eye phenotype by increasing ommatidial disorganization (Figure 4B). Overexpression of Drep-2 could suppress the rCGG-induced eye phenotype (Figure 4C). Flies carrying the Drep-2 mutation alone or Drep-2 overexpression alone displayed normal eyes (Figure 4D and 4E). We also found a heterozygous loss-of-function mutant of Vimar that aggravated the rCGG_90_ eye phenotype (Figure 4F). Eyes of control flies carrying the same Vimar mutation but no rCGG_90_ repeats are normal (Figure 4G). These data together indicate that Drep-2 and Vimar could modulate rCGG-mediated neurodegeneration.

**Table 1:** Mutant alleles corresponding to the mRNA targets of miR-277 used for genetic screen.

Gene	Alleles	Potential function
Polypeptide GalNAc transferase 5 (Pgant5)	*pgant5^B480^*	Oligosaccharide biosynthetic process
Frizzled (Fz)	*fz^EY03114^*	Wnt receptor activity
CG5599	*CG5599^KG02236^*	Dihydrolipoamide branched chain acyltransferase activity
Netrin-B (NetB)	*NetB^KG03586^*	Motor axon guidance, salivary gland boundary specification
CG32767	*CG32767^BG01357^*	Nucleic acid binding
Decapping protein 2 (Dcp2)	*Dcp2^BG01766^*	RNA binding, hydrolase activity, manganese ion binding
Belphegor (Bor)	*bor^c05496^*	Nucleoside-triphosphatase activity, ATP binding
CG3267	*CG3267^e02164^*	Methylcrotonoyl-CoA carboxylase activity, CoA carboxylase activity
DNA fragmentation factor-related protein 2 (Drep-2)	*Drep-2^KG02396^* *Drep-2^d00223^*	Apoptosis, neurogenesis
GTPase-activating protein 1 (Gap1)	*Gap1^B2^*	Ras GTPase activator activity
G protein oα 47A (G-oα47A)	*G-oα47A^01^*	GTP binding
CG1673	*CG1673^EP1023^*	Branched-chain-amino-acid transaminase activity
Abrupt (Ab)	ab^k02807^	Sequence-specific DNA binding transcription factor activity
Visceral mesodermal armadillo-repeats (Vimar)	*vimar^k16722^*	Ral GTPase binding, modulator of mitochondrial function
Scribbled (scrib)	*scrib^j7B3^*	Protein binding, post-embryonic organ morphogenesi
Flotillin 2 (Flo-2)	*Flo-2^BG01945^*	Cell adhesion
Synaptotagmin β (Sytβ)	*Sytβ^BG02150^*	Calcium-dependent phospholipid binding
Starry night (stan)	*stan^f00907^*	Receptor signaling protein activity
Scully (scu)	*scu^f05851^*	NADP+ activity
CG15093	*CG15093^d04924^*	Cellular amino acid metabolic process
CG18265	*CG18265^EY11347^*	Zinc ion binding
Gliotactin (Gli)	*Gli^EY11600^*	Carboxylesterase activity
Elbow B (Elb)	*elB^3^*	Zinc ion binding, nucleic acid binding
Cirl	*Cirl^EY12930^*	Latrotoxin receptor activity
Task6	*Task6^EY23668^*	Potassium channel activity

### MiR-277 regulates the expression of Drep-2 and Vimar post-transcriptionally

**Figure 5:**
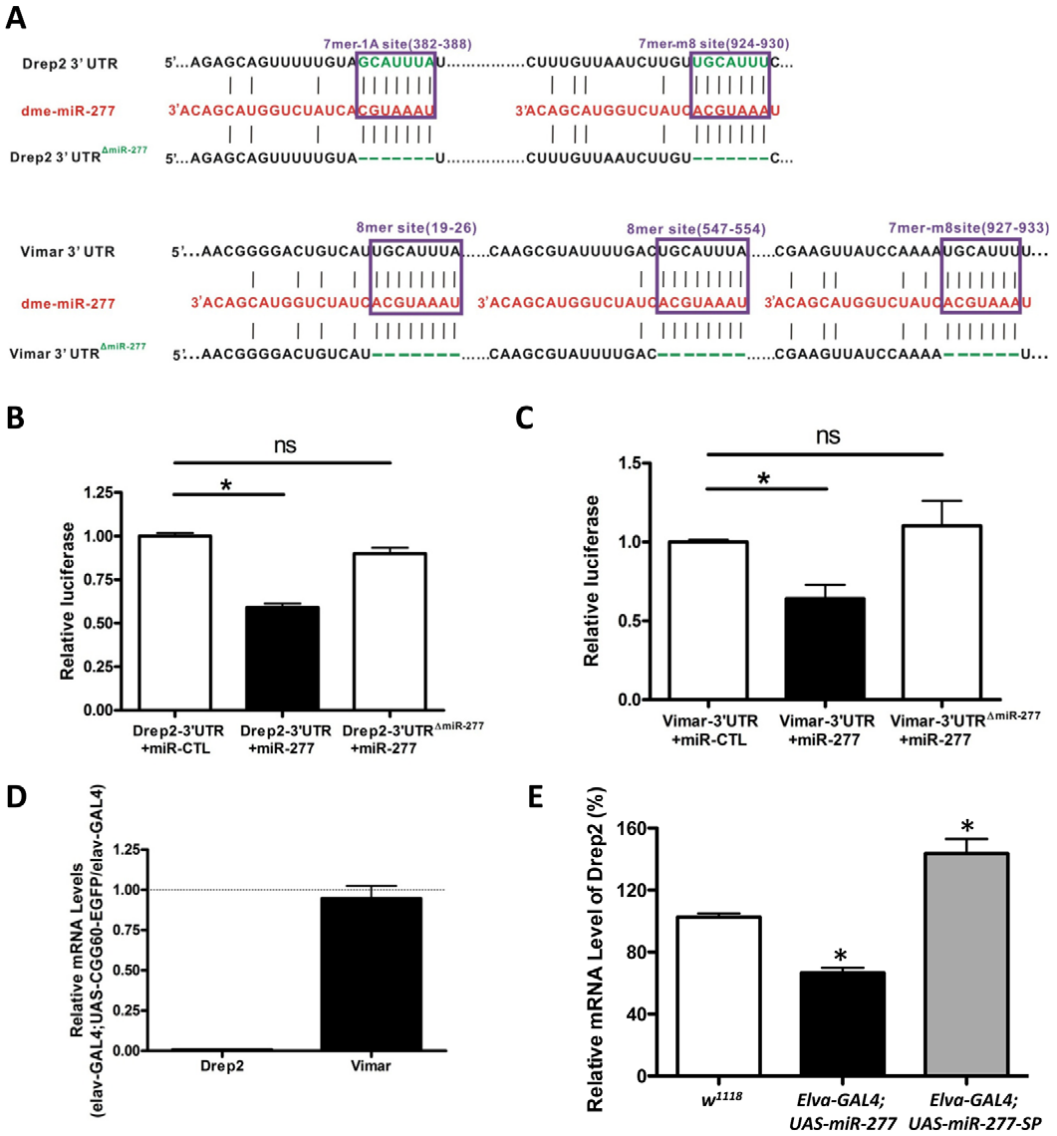
MiR-277 regulates the expression of Drep-2 and Vimar post-transcriptionally. A. The two miR-277 target sites in the Drep-2 3′-UTR and three miR-277 target sites in the Vimar 3′-UTR were predicted by TargetScan. We created the mutant Drep-2 3′-UTR (Drep-2 3′-UTR^ΔmiR-277^) with two miR-277 binding sites deleted and the mutant Vimar 3′-UTR (Vimar 3′ UTR^ΔmiR-277^) with three miR-277 binding sites deleted, as shown. B. The expression of a luciferase reporter gene containing the Drep-2 3′-UTR was suppressed by miR-277 in HEK293FT cells. The suppression is specific to the miR-277 seed region within the Drep-2 3′-UTR, as deletion of the miR-277 target sites in the Drep-2 3′-UTR alleviated repression by miR-277. (*-P<0.05; ns-P>0.05). C. Similar miR-277-mediated suppression was observed with Vimar 3′-UTR constructs. D. Endogenous Drep-2 mRNA expression was significantly reduced in rCGG repeat flies relative to control flies (elav-GAL4), whereas no obvious change of Vimar mRNA expression was observed in the rCGG repeat flies compare to control flies. E. Endogenous Drep-2 mRNA expression was significantly altered in the flies expressing either miR-277 or miR-277 SP, *-P<0.05.

Since both Drep-2 and Vimar were predicted to be regulated by miR-277, by introducing 3′-UTR dual luciferase assays, we tested whether miR-277 could indeed target to Drep-2 or Vimar. We cloned the 3′-UTR of Drep-2 or Vimar containing the predicted miR-137 target sites from fly cDNA into a dual luciferase (R-Luc and F-Luc) reporter construct, allowing for the assessment of protein translation of these targets regulated via their 3′-UTR (Figure 5A). These 3′-UTR dual constructs were transfected into HEK293 cells. We found that overexpression of miR-277 could suppress the R-Luc activity at 48 h post-transfection (Figure 5B and 5C). Furthermore, when we mutated the seed regions of miR-277 located within the Drep2-3′-UTR reporter and Vimar-3′-UTR reporter, we saw that the mutation alleviated the miR-277-mediated suppression of luciferase activity (Figure 5A–5C), suggesting the action of miR-277 is specific to the miR-277 seed region within the Drep-2 3′-UTR and Vimar 3′-UTR. Together, these data demonstrate that the effect of miR-277 on Drep-2 and Vimar expression is repressive and specific. Importantly, they also suggest that Drep-2 and Vimar are the functional targets of miR-277.

Next we went on to examine the steady-state levels of Drep-2 and Vimar mRNA in rCGG repeat flies (*elav-GAL4;UAS-CGG_60_-EGFP*), in which the expression of miR-277 is increased. We saw a significant reduction of endogenous Drep-2 mRNA in rCGG repeat flies relative to control flies (*elav-GAL4*), whereas the Vimar mRNA expression in rCGG repeat flies remained similar to control flies (*elav-GAL4*) (Figure 5D). Furthermore, ectopic expression of miR-277 or miR-277 sponge could alter the endogenous mRNA level of Drep2 while have no apparent effect on Vimar mRNA level (Figure 5E). These observations suggest that miR-277 could regulate Drep-2 and Vimar mRNAs differentially, with miR-277 regulating the expression of Drep-2 mainly at the mRNA level, and Vimar via translational suppression instead.

### Expression of miR-277 is regulated by the rCGG repeat-binding protein hnRNP A2/B1

**Figure 6:**
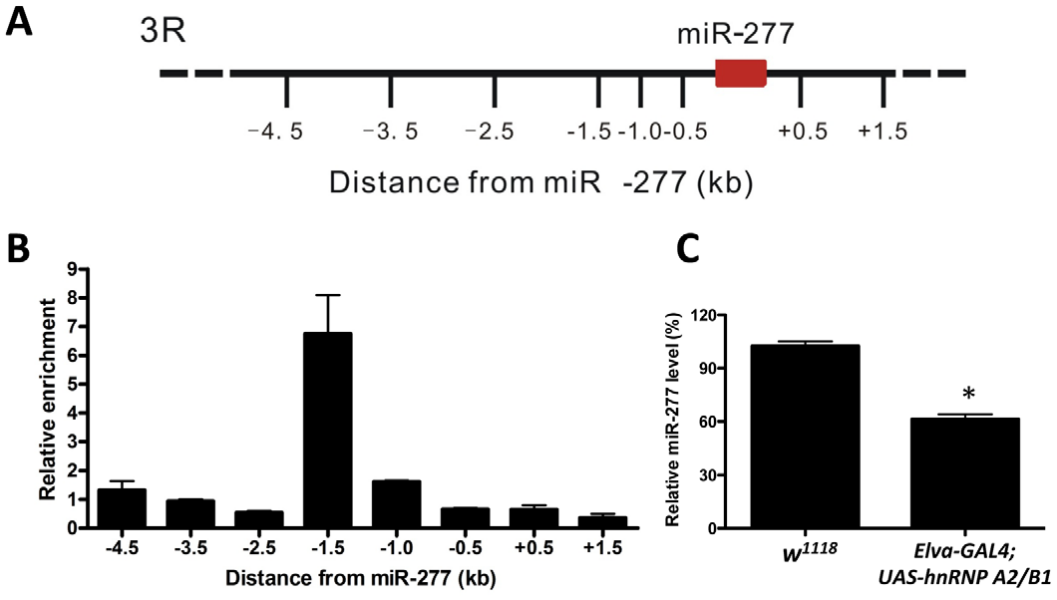
Expression of miR-277 is regulated by hnRNPA2/B1. A. Schematic of the six kb proximal to miR-277 on chromosome 3R assayed in ChIP experiments. B. HnRNP A2/B1-specific ChIP assay indicates the enrichment of DNA 1.5 kb upstream of the miR-277 genomic locus. Relative enrichment is calculated relative to IgG-only nonspecific control and normalized to the empty vector. Error bars indicate mean ± SEM. C. Ectopic expression of hnRNP A2/B1 in fly brain could suppress the expression of miR-277, *-P<0.05.

Two rCGG repeat-binding proteins, Pur α and hnRNP A2/B1, were previously found to bind rCGG repeats directly and modulate rCGG-mediated neuronal toxicity [21], [22]. Intriguingly, recent studies have shown that multiple heterogeneous nuclear ribonucleoproteins (hnRNPs) could interact with heterochromatin protein 1 (HP1) to bind to genomic DNA and modulate heterochromatin formation [46]. Thus we tested whether hnRNP A2/B1 could interact directly with genomic regions proximal to miR-277. We performed hnRNP A2/B1-specific chromatin immunoprecipitation (ChIP) followed by real-time quantitative PCR across a six-kb region surrounding miR-277. Immunoprecipitation of chromatin chemically cross-linked to DNA with an hnRNP A2/B1-specific antibody demonstrated that a region 1.5 kb upstream of miR-277 was enriched ∼seven-fold relative to IgG control and adjacent regions (Figure 6A and 6B). Furthermore, the ectopic expression of hnRNP A2/B1 in fly brain could reduce the expression of miR-277 (Figure 6C). These results together suggest that hnRNP A2/B1 could directly bind to the upstream region of miR-277 and regulate its expression. In the presence of fragile X premutation rCGG repeats, hnRNP A2/B1 will be sequestered, leading to the de-repression of the miR-277 locus.

## Discussion

**Figure 7:**
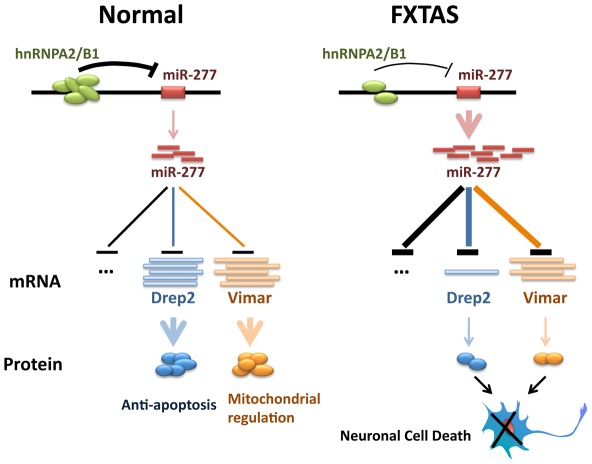
Proposed model of miR-277 modulating fragile X premutation rCGG repeat-mediated neurodegeneration. HnRNP A2/B1, an rCGG repeat-binding protein, could be sequestered by fragile X premutation rCGG repeats from their normal cellular functions. Sequestration of hnRNP A2/B1 could de-repress the expression of miR-277. MiR-277 could recognize and bind to the 3′-UTR of its mRNA targets, such as Drep-2 and Vimar, to induce their mRNA degradation or translational suppression. Misregulation of Drep-2 or Vimar, which are involved in cell apoptosis or mitochondrial regulation, could lead to the neuronal cell death associated with FXTAS.

Fragile X-associated tremor/ataxia syndrome (FXTAS) is a neurodegenerative disorder that afflicts fragile X syndrome premutation carriers, with earlier studies pointing to FXTAS as an RNA-mediated neurodegenerative disease. Several lines of evidence suggest that rCGG premutation repeats may sequester specific RNA-binding proteins, namely Pur α, hnRNP A2/B1, and CUGBP1, and reduce their ability to perform their normal cellular functions, thereby contributing significantly to the pathology of this disorder [15], [21], [22]. The miRNA pathway has been implicated in the regulation of neuronal development and neurogenesis [32], [47]–[49]. A growing body of evidence has now revealed the role of the miRNA pathway in the molecular pathogenesis of neurodegenerative disorders [35]. Here we demonstrate that specific miRNAs can contribute to fragile X rCGG repeat-mediated neurodegeneration by post-transcriptionally regulating target mRNAs that are involved in FXTAS. We show that miR-277 plays a significant role in modulating rCGG repeat-mediated neurodegeneration. Overexpression of miR-277 enhances rCGG repeat-induced neuronal toxicity, whereas blocking miR-277 activity could suppress rCGG repeat-mediated neurodegeneration. Furthermore, we identified Drep-2 and Vimar as the functional miR-277 targets that could modulate rCGG repeat-induced neurodegeneration. Finally, we show that hnRNP A2/B1, an rCGG repeat-binding protein, can directly regulate the expression of miR-277. Our biochemical and genetic studies demonstrate a novel miRNA-mediated mechanism involving miR-277, Drep-2, and Vimar in the regulation of neuronal survival in FXTAS (Figure 7).

Several lines of evidence from studies in mouse and *Drosophila* models strongly support FXTAS as an RNA-mediated neurodegenerative disorder caused by excessive rCGG repeats [11], [15], [17]–[19]. The current working model is that specific RNA-binding proteins could be sequestered by overproduced rCGG repeats in FXTAS and become functionally limited, thereby contributing to the pathogenesis of this disorder [15], [17], [19], [20]. Three RNA-binding proteins are known to modulate rCGG-mediated neuronal toxicity: Pur α, hnRNP A2/B1, and CUGBP1, which bind rCGG repeats either directly (Pur α and hnRNP A2/B1) or indirectly (CUGBP1, through the interaction with hnRNP A2/B1) [21], [22]; how the depletion of these RNA-binding proteins could alter RNA metabolism and contribute to FXTAS pathogenesis has thus become the focus in the quest to understand the molecular pathogenesis of this disorder. Nevertheless, the data we present here suggest that the depletion of hnRNP A2/B1 could also directly impact the transcriptional regulation of specific loci, such as miR-277. We know that hnRNPs can interact with HP1 to bind to genomic DNA and modulate heterochromatin formation [46]. Our results indicate that hnRNP A2/B1 could participate in the transcriptional regulation of miR-277; however, it remains to be determined whether other loci could be directly regulated by hnRNP A2/B1, as well. Identifying those loci will be important to better understand how the depletion of rCGG repeat-binding proteins could lead to neuronal apoptosis.

In recent years, several classes of small regulatory RNAs have been identified in a range of tissues and in many species. In particular, miRNAs have been linked to a host of human diseases. Some evidence suggests the involvement of miRNAs in the emergence or progression of neurodegenerative diseases. For example, accumulation of nuclear aggregates that are toxic to neurons have been linked to many neurodegenerative diseases, and miRNAs are known to modulate the accumulation of the toxic proteins by regulating either their mRNAs or the mRNAs of proteins that affect their expression. Moreover, miRNAs might contribute to the pathogenesis of neurodegenerative disease downstream of the accumulation of toxic proteins by altering the expression of other proteins that promote or inhibit cell survival [35]. Our genetic modifier screen revealed that miR-277 could modulate rCGG repeat-mediated neurodegeneration. By combining our genetic screen and reporter assays, we identified Drep-2 and Vimar as the functional targets of miR-277 that could modulate rCGG-mediated neurodegeneration. The closest ortholog of miR-277 in human is miR-597 based on the seed sequence. It would be interesting to further examine the role of miR-597 in FXTAS using mammalian model systems.

Drep-2 is associated with the chromatin condensation and DNA fragmentation events of apoptosis [50], [51]. Drep-2 is one of four *Drosophila* DFF (DNA fragmentation factor)-related proteins. While Drep-1 is a *Drosophila* homolog of DFF45 that can inhibit CIDE-A mediated apoptosis [50]. Drep-2 has been shown to interact with Drep-1 and to regulate its anti-apoptotic activity [50]. Vimar is a Ral GTPase-binding protein that has been shown to regulate mitochondrial function via an increase in citrate synthase activity [52]. In the presence of fragile X premutation rCGG repeats, overexpression of miR-277 will suppress the expression of both Drep-2 and Vimar, thereby altering anti-apoptotic activity as well as mitochondrial functions, which have been linked to neuronal cell death associated with neurodegenerative disorders in general (Figure 7). Interestingly, we saw a significant reduction of Drep-2 mRNA in the flies expressing rCGG repeats, while Vimar mRNA levels remained similar to control flies. This observed difference may be due to the fact that miRNA could be involved in different modes of action, including mRNA cleavage, translational inhibition and mRNA decapping/deadenylation its target mRNAs [23], [24].

In summary, here we provide both biochemical and genetic evidence to support a role for miRNA and its selective mRNA targets in rCGG-mediated neurodegeneration. Our results suggest that sequestration of specific rCGG repeat-binding proteins can lead to aberrant expression of selective miRNAs that could modulate the pathogenesis of FXTAS by post-transcriptionally regulating the expression of specific mRNAs involved in this disorder. Identification of these miRNAs and their targets could reveal potential new targets for therapeutic interventions to treat FXTAS, as well as other neurodegenerative disorders.

## Materials and Methods

### *Drosophila* genetics

All flies were maintained under standard culture conditions. The rCGG repeat transgenic flies (*UAS-CGG_60_-EGFP* and *UAS-CGG_90_-EGFP*) were generated in our lab as described previously [15]. Flies mutant in genes coding for different candidate miR-277 targets were obtained from Bloomington Stock Center. The UAS-miR-277-Sponge transgenic flies were generated as described previously [43]. We introduced 10 repetitive miR-277 sponge sequences (TGTCGTACCAGGCGTGCATTTA) with a 4-nt linker between each repeat downstream of EGFP in a pUASP expression vector. Similarly a scramble control construct (GTTCACGGATAGTGCCTGTACT) was generated as well. Both constructs were confirmed by DNA sequencing and then injected in the *w^1118^* strain by standard methods.

### Relative quantification of mature miRNAs by TaqMan miRNA real-time PCR

Total RNAs were isolated from the control (elav-GAL4) and rCGG_60_ fly heads using Trizol. TaqMan MicroRNA Assays detecting 72 known individual *Drosophila* miRNAs were obtained from ABI (ABI). cDNA was prepared with High-Capacity cDNA Reverse Transcription Kits (ABI; Cat#437496). The 15-µl reverse transcription reactions consisted of 10 ng of total RNA, 5 U MultiScribe Reverse Transcriptase, 0.5 mM of each dNTP, 1× reverse transcription buffer, 4 U RNase inhibitor, and nuclease-free water. This was performed at 16°C for 30 min and at 42°C for 30 min, terminated at 85°C for 5 min and 4°C until use in TaqMan assays. For real-time PCR of TaqMan MicroRNA Assays, we used 0.5 ul 20×TaqMan MicroRNA Assay Primer, 1.33 ul undiluted cDNA, 5 ul 2×TaqMan Universal PCR Master Mix, 3.17 ul nuclease-free water. Each PCR reaction was performed in triplicate with MicroAmp optical 96-well plates using a 7500 Fast Real-Time PCR System (ABI), with reactions incubated at 95°C for 10 min, followed by 40 cycles of 95°C for 15 s, and 60°C for 1 min. Fluorescence readings were taken during the 60°C step. RQs were calculated using the ΔΔCt method, with 2S RNA TaqMan miRNA control assay as the endogenous control, and calibrated to the control samples.

### Quantitative RT–PCR

The fly heads from control (*elav-Gal4*) and rCGG_60_ flies were collected. Trizol (Invitrogen; Cat# 15596-026) was used to isolate total RNA from each genotype. RNA samples were reverse-transcribed into cDNA with oligo(dT)_20_ and SuperScript III (Invitrogen; Cat#18080051). Real-time PCR was performed with gene-specific primers and Power SYBR Green PCR Master Mix (Applied Biosystems; Cat# 4367660) using the 7500 Standard Real-Time PCR System (Applied Biosystems). RpL32 (Qiagen; Cat# QT00985677) was used as an endogenous control for all samples. Primers for Drep2 and Vimar transcripts were designed using Primer Express 3.0 software (Applied Biosystems) and were as follows. Drep2: forward, 5′-TGGAACGCCTCAACTCCAA-3′; and reverse, 5′-TCGGACTCGCGATCCAA-3′. Vimar: forward, 5′-GCACCCGCCGAACAGA-3′; and reverse, 5′-TGCGATCGTAGTCTTGCGTTA -3′. All real-time PCR reactions were performed in triplicate, and RQs were calculated using the ΔΔCt method, with calibration to control samples.

### 3′-UTR dual luciferase assays of candidate miR-277 target mRNA

Drep-2 3′-UTR and Vimar 3′-UTR sequences were PCR-amplified directly from *w^1118^* fly brain first-strand cDNA generated from 5 ug TRIZOL-isolated total RNA using oligo-dT SuperScript III reverse transcription according to the manufacture's protocol (Invitrogen; Cat. #1808-093). The PCR products were then cloned into psiCHECK-2 dual luciferase vector (Promega; Cat# C8021). The miR-277 target sites in the Drep-2 3′-UTR and Vimar 3′-UTR were deleted using QuikChange Site-Directed Mutagenesis Kits (Stratagene; Cat. #2000518). Target sites deletions were verified by Genewiz sequencing. Briefly, 293FT cells were co-transfected by Attractene transfection reagent (Qiagen; Cat. #301005) with psiCHECK-2-3′UTR or psiCHECK-2-3′ UTRΔmiR-277 and miR-277 duplex RNA (Qiagen; Cat# MSY0000338) or control miRNA duplex (Qiagen; Cat# 1027280). All co-transfections used a total of 600 ng of plasmid DNA and 120 nmol of duplex RNA. Luciferase expression was detected using the Dual-Luciferase Reporter 1000 System (Promega; Cat# E1980) according to the manufacturer's instructions. At 48 h after transfection, R-Luc activity was normalized to F-Luc activity to account for variation in transfection efficiencies, and miR-277-mediated knockdown of R-Luc activity was calculated as the ratio of normalized R-Luc activity in the miR-277 duplex treatments to normalized R-Luc activity in the negative control duplex treatments. Luciferase experiments were repeated three times.

### Chromatin immunoprecipitation (ChIP)

ChIP was performed using a ChIP Assay Kit (Millipore). S2 cells were cross-linked with 1% formaldehyde (Sigma-Aldrich) for 10 min at room temperature. Chromatin was fragmented to an average size of 500 bp by sonication (Sonicator 3000; Misonix) and immunoprecipitated with anti-Flag M2 antibody (sigma). Immunoprecipitated and purified DNA fragments were diluted to 1 ng/µl in nuclease-free water. We used 8 ng of DNA in 20-µl SYBR Green real-time PCR reactions consisting of 1× Power SYBR Green Master Mix and 0.5 µM forward and reverse primers. Reactions were run on an SDS 7500 Fast Instrument (Applied Biosystems). Primers were designed using Primer Express 3.0 software (Applied Biosystems) and were as follows. 4.5 kb upstream: forward, 5′-CAGAAAACAGGCGTGCAAAC; and reverse, 5′-GAATTTGCATTGGCTTTGGAA. 3.5 kb upstream: forward, 5′- TTACAATTGGATGGGCTTCGT; and reverse, 5′-AAGCTGACGGCCTGACTAAAAA. 2.5 kb upstream: forward, 5′-GTTGGCTGCTGCGTCAATT; and reverse, 5′- GCCCCAGCGGCATTTATA. 1.5 kb upstream: forward, 5′- TTCTGGCACTGGCAGCTTT; and reverse, 5′- CATCGTGCTGGCCAACAC. 1.0 kb upstream: forward, 5′-TGTACGGGCATGTGTATGCA; and reverse, 5′- TCAACGAACACGCTGCGTAT. 0.5 kb upstream: forward, 5′- GGGCATTTTCATTTCATTCCA; and reverse, 5′- CGGGCAGCGTAATTTAAGCT. 0.5 kb downstream: forward, 5′- CGCCCACAAGAGCTTTTGA; and reverse, 5′- TTTCCACGGTATGCTGCTTTT. 1.5 kb downstream: forward, 5′- CGTTTCCATTTAGTTGGATTTTTGT; and reverse, 5′- GGCAAACCACACATTTTAACATACA. DNA relative enrichment was determined by taking the absolute quantity ratios of specific IPs to nonspecific IPs (normal mouse IgG only), IP/IgG, and normalizing to control (pUAST only). Independent chromatins were prepared for all ChIP experiments, and real-time PCR reactions were performed in triplicate for each sample on each amplicon.

### Scanning electron microscopy

For scanning electron microscopy (SEM) images, whole flies were dehydrated in gradient concentration ethanol (25%, 50%, 75%, 100%), dried with hexamethyldisilazane (Sigma; Cat# 16700), and analyzed with an ISI DS-130 LaB6 SEM/STEM microscope.
